# Aquatic adaptation and the evolution of smell and taste in whales

**DOI:** 10.1186/s40851-014-0002-z

**Published:** 2015-02-13

**Authors:** Takushi Kishida, JGM Thewissen, Takashi Hayakawa, Hiroo Imai, Kiyokazu Agata

**Affiliations:** Graduate School of Science, Kyoto University, Kitashirakawa Oiwake-cho, Sakyo, Kyoto 606-8502 Japan; Department of Anatomy and Neurobiology, Northeast Ohio Medical University, 4209 State Route 44, Rootstown, OH 44272 USA; Primate Research Institute, Kyoto University, Kanrin, Inuyama, Aichi 484-8506 Japan; Japan Society for the Promotion of Science, Kojimachi, Chiyoda, Tokyo 102-0083 Japan; Present affiliation: Wildlife Research Center, Kyoto University, 2-24 Tanaka Sekiden-cho, Sakyo, Kyoto 606-8203 Japan

**Keywords:** Antarctic minke whale genome, Archaeoceti, Cetacea, Chemoreception, Olfactory bulb

## Abstract

**Introduction:**

While olfaction is one of the most important senses in most terrestrial mammals, it is absent in modern toothed whales (Odontoceti, Cetacea). Furthermore, behavioral evidence suggests that gustation is very limited. In contrast, their aquatic sistergroup, baleen whales (Mysticeti) retain small but functional olfactory organs, and nothing is known about their gustation. It is difficult to investigate mysticete chemosensory abilities because experiments in a controlled setting are impossible.

**Results:**

Here, we use the functional regionalization of the olfactory bulb (OB) to identify the loss of specific olfactory functions in mysticetes. We provide the whole-genome sequence of a mysticete and show that mysticetes lack the dorsal domain of the OB, an area known to induce innate avoidance behavior against odors of predators and spoiled foods. Genomic and fossil data suggest that mysticetes lost the dorsal domain of the OB before the Odontoceti-Mysticeti split. Furthermore, we found that all modern cetaceans are revealed to have lost the functional taste receptors.

**Conclusion:**

These results strongly indicate that profound changes in the chemosensory capabilities had occurred in the cetacean lineage during the period when ancestral whales migrated from land to water.

**Electronic supplementary material:**

The online version of this article (doi:10.1186/s40851-014-0002-z) contains supplementary material, which is available to authorized users.

## Introduction

Terrestrial mammals usually have a well-developed sense of smell that can detect various odors using four kinds of G-protein coupled receptors (GPCRs) encoded by different multigene families to each other: olfactory receptors (ORs), trace amine-associated receptors (TAARs) and two types of vomeronasal receptors (V1Rs and V2Rs) [1]. But this sense was greatly reduced in the ancestors of modern cetaceans [2]. Modern cetaceans lack a large number of *OR* genes [3-5], and odontocetes lost the nervous system structures that mediate olfaction, such as the olfactory tract, olfactory bulb (OB) and cranial nerve I [6]. In addition to the four olfactory GPCRs, two GPCR families are involved in mammalian gustation: TAS1R (taste receptor type 1, the sweet and umami taste receptor) and TAS2R (taste receptor type 2, the bitter taste receptor) [1]. Most of the taste receptor genes have also been lost from dolphin genomes [7,8], though behavioral tests indicate that dolphins can detect several kinds of flavorants [9]. In contrast, mysticetes retained these anatomical structures, although they are small [10], and it has been suggested that mysticetes use olfaction in foraging [11]. Like those of terrestrial mammals, mysticetes’ olfactory nerves are concentrated in their nasal cavities [10], and their nasal passages remain filled with air when they dive and keep water out, indicating that mysticetes can smell in air but not underwater. Unfortunately, no mysticete species are kept in laboratories or aquariums, meaning that experiments in a controlled setting are impossible, and thus it is still a mystery how mysticetes use olfaction for their fully aquatic life. Regarding taste, most of their taste receptors have been lost [12,13], but it is not clear whether the remaining receptors are still functional or not.

Olfaction has been studied in laboratory mammals: olfactory sensory neurons (OSNs) are located in the olfactory epithelium of the nasal cavity and each OSN expresses only one chemosensory receptor gene [14]. The axons of the OSNs that express the same receptors converge to a set of glomeruli in the OB that are in a distinct topographic region of the OB [15]. Thus, odorous information received in the olfactory epithelium is converted to topographical maps of activated glomeruli of the OB. The glomerular layer of the OB can be divided into two non-overlapping areas, a dorsal domain (D domain) and a ventral domain (V domain) based on the expression patterns of several domain-specific marker genes [16,17]. D domain-ablated mice (ΔD mice) fail to show innate avoidance behavior against predator odors and spoiled smells [16].

We previously studied the anatomy and histology of the OB in a single mysticete (bowhead whale *Balaena mysticetus*) [10]. Olfactory nerves enter the OB from the ventral side in these mysticetes, and connect to glomeruli located on the ventral side. However, unlike OBs in most other mammals, dorsal OSN axons and glomeruli are absent or nearly absent. This distribution of glomeruli resembles that of ΔD mice, and this led us to hypothesize that mysticetes lack the D domain of the OB.

To test this hypothesis, we applied a whole-genome shotgun strategy and *de Bruijn* graph-based algorithms to sequence and assemble the Antarctic minke whale (*Balaenoptera bonaerensis*, Mysticeti) genome, and compared it to a dolphin (an odontocete) and a cow (an artiodactyl, the cetacean sister group). In addition, we investigated fossils to understand the evolution of whale OB from the morphological aspects. Genetic evidences about mysticete gustation are also examined based on the genome assembly.

## Materials and methods

### Genome sequencing and assembly

Muscle tissue of Antarctic minke whale was purchased from a fish market in Japan, and the genomic DNA was extracted following the protocol of our previous work [4]. A paired-end sequencing library with average insert size of 330 bp was constructed and sequenced on an Illumina HiSeq2000 sequencer, and then assembled into scaffolds using PLATANUS assembler [18] ver. 1.2.1. Details about genome sequencing and *de novo* assembly are described in Additional file 1 §1. The Antarctic minke whale genome assembly thus obtained was named KUjira_1.0.

Cow (*Bos taurus*, Artiodactyla) genome assembly (UMD_3.1 assembly) [19] were downloaded from the GenBank FTP site (ftp://ftp.ncbi.nlm.nih.gov/genbank/genomes/Eukaryotes/vertebrates_mammals/Bos_taurus/Bos_taurus_UMD_3.1/). Bottlenose dolphin (*Tursiops truncatus*, Odontoceti) genome assembly (Ttru_1.4 assembly) [20] were also downloaded from the GenBank FTP site (ftp://ftp.ncbi.nlm.nih.gov/genbank/genomes/Eukaryotes/vertebrates_mammals/Tursiops_truncatus/Ttru_1.4/).

### Olfaction-related genes in the cow genome

The loci of the *OMACS*, *NQO1* and *OCAM* genes in the cow UMD_3.1 genome assembly follow NCBI reference sequence (RefSeq) annotations. The gene ID of each gene is as follows: *OMACS*, 100299006; *NQO1*, 519632; *OCAM*, 535613. We confirmed the RefSeq annotations by comparing translated amino acid sequences with those of other mammals. The amino acid sequences of 15 mouse TAARs (TAAR1 (GenBank accession no. NP_444435.1), TAAR2 (NP_001007267.1), TAAR3 (NP_001008429.1), TAAR4 (NP_001008499.1), TAAR5 (NP_001009574.1), TAAR6 (NP_001010828.1), TAAR7a (NP_001010829.1), TAAR7b (NP_001010827.1), TAAR7d (NP_001010838.1), TAAR7e (NP_001010835.1), TAAR7f (NP_001010839.1), TAAR8a (NP_001010830.1), TAAR8b (NP_001010837.1), TAAR8c (NP_001010840.1), TAAR9 (NP_001010831.1)) and six human TAARs (TAAR2-1 (NP_001028252.1), TAAR2-2 (NP_055441.2), TAAR5 (NP_003958.2), TAAR6 (NP_778237.1), TAAR8 (NP_444508.1), TAAR9 (NP_778227.3)) were used as queries and TAAR sequences were searched against the cow genome assembly using TBLASTN program ver. 2.2.25 [21] with e-value cutoff of <1e-20 and without filtering query sequences. All overlapping sequences of hits with the same orientations were merged. The sequences thus obtained were searched against the mouse protein database (downloaded from the following URL on 14/Oct/2011: http://www.ncbi.nlm.nih.gov/protein/?term=%22Mus+musculus%22%5Bporgn%3A__txid10090%5D) using FASTY program ver. 35.04 [22] and the sequence was discarded if its best hit was not a *TAAR* gene. Then we aligned all the remaining sequences using the L-INS-i program in the MAFFT package ver. 6.240 [23,24] and looked for the initiation and termination codons. If we could not find initiation and/or termination codons in a sequence, we extended the sequence in the 5’ and/or 3’ direction to find them. If a sequence was interrupted by premature stop codon(s) and/or frame shift(s), or if it lacked one or more trans-membrane (TM) regions completely, the sequence was judged to be a functionless pseudogene. As a result, 17 intact *TAAR* genes and 14 pseudoegenes were found. The classification of intact cow *TAAR* genes into TAAR1-9 follows the phylogenetic tree shown in Additional file 1 §3. Deduced amino acid sequences of 142 class I and 828 class II intact OR genes were retrieved from Niimura and Nei [25].

### Olfaction-related genes in the whale and dolphin genomes

**Figure 1:**
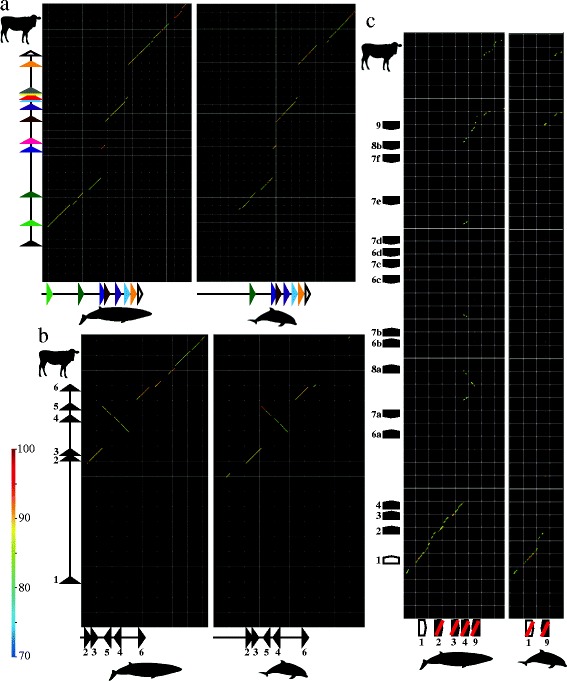
**Dot-plot comparisons between Antarctic minke whale (horizontal, left) or dolphin (horizontal, right) and cow (vertical) sequences.** Color scale bar indicates sequence similarity (%) of each dot. **a**. Comparisons of the genomic region where the *OMACS* gene is encoded. Cow sequence: chr. 25 (18,535,000-18,568,000 bp). Whale: scaffold100261 (1–18,000 bp, complement). Dolphin: scaffold4608 (95,000-115,000 bp, complement). The *OMACS* gene consists of 13 exons, and the position and the coding direction of each exon is shown as a triangle with an exon-specific color. Whale and dolphin have lost the genomic regions where the 5^th^, 9^th^, 10^th^ and 11^th^ exons are encoded. In addition, dolphin has lost the 1^st^ and 2^nd^ exons. Whale’s 1^st^ exon is not included in this scaffold (see Additional file 1 for detail). Mesh size, 1 kbp × 1 kbp. **b**. Comparison of the genomic region where the *NQO1* gene is encoded. Cow sequence: chr. 18 (36,908,355-36,927,688 bp). Whale: scaffold73885. Dolphin: scaffold317 (290,000-300,000 bp). The *NQO1* gene consists of six exons, and the position and the coding direction of each exon are shown as a triangle with an exon number. Genomic inversion was confirmed in the whale and dolphin genomes around the region where the 4^th^ and 5^th^ exons are encoded. Whale’s 1^st^ exon is not included in this scaffold (see Additional file 1 for detail). Mesh size, 1 kbp × 1 kbp. **c**. Comparison of the genomic region where the *TAAR* gene cluster is located. Cow sequence: chr. 9 (71,400,000-71,850,000 bp, complement). Whale: scaffold12993. Dolphin: scaffold181 (230,000-270,000 bp, complement). Positions and coding directions of *TAAR1-9* genes are shown. Pseudogenes are indicated by red oblique lines (Cow *TAAR* pseudogenes are not shown). Mesh size, 10 kbp × 10 kbp.

For the multi-exon *OMACS*, *NQO1* and *OCAM* genes, we used the DNA sequence of each exon of the corresponding cow genes as a query and searched against the minke whale and dolphin genome assemblies using BLASTN with e-value cutoff of <1e-20 and without filtering query sequences. The sequences thus obtained were searched against the cow genome assembly using BLASTN and the sequence was discarded if its best hit was not its query. Several exons of the *OMACS* gene cannot be found following this method, and therefore we compared the genomic regions encoding other exons with that of cow genome in order to confirm that these missing exons are actually deleted from whale and dolphin genomes. For dot-plot comparisons, GenomeMatcher ver. 1.75 [26] was used with default settings, and the bl2seq [27] option was chosen to output figures (Figure 1). We followed the same methods which we used to identify cow *TAAR* genes to identify whale and dolphin *TAAR*, *OR* and *V1R* genes, using the query amino acid sequences as follows: 17 intact cow TAARs for searching *TAAR* sequences, 970 intact cow ORs (142 class I and 828 class II) for *OR* sequences and 32 cow intact V1Rs identified by Grus *et al.* [28] for *V1R* sequences. Because of fragmented scaffolds, we could not find initiation and/or termination codons of several sequences which were not judged to be pseudogenes. We labeled such sequences as truncated genes. Under these criteria, we found 324 *OR* genes (60 intact, 19 truncated and 245 pseudo) and 34 V1R genes (two intact and 32 pseudo) in the KUjira_1.0 assembly, and 166 OR genes (twelve intact, two truncated and 152 pseudo) and 18 V1R genes (one intact and 17 pseudo) in the Ttru_1.4 assembly. However, only five *TAAR* genes and pseudogenes were found from the KUjira_1.0 assembly and two in the Ttru_1.4 assembly. Therefore, we compared the genomic regions encoding a cluster of *TAAR* genes with that of the cow genome using the GenomeMatcher program in order to confirm that the missing *TAAR* genes are actually deleted from whale and dolphin genomes. In the case of multi-exon *V2R* genes, we also followed the same methods as described above but we searched only 3^rd^ exons using 3^rd^ exons of 79 intact rat V2Rs identified by Young and Trask [29] as queries. As a result, we could not find any sequences in the Ttru_1.4 assembly but we found one sequence in the KUjira_1.0 assembly. However, premature stop codons interrupt its open reading frame. Therefore, we conclude that this exon is a part of a functionless pseudogene and that neither whale nor dolphin possesses intact *V2R* genes.

### Classification of cetacean OR genes into class I/class II

**Figure 2:**
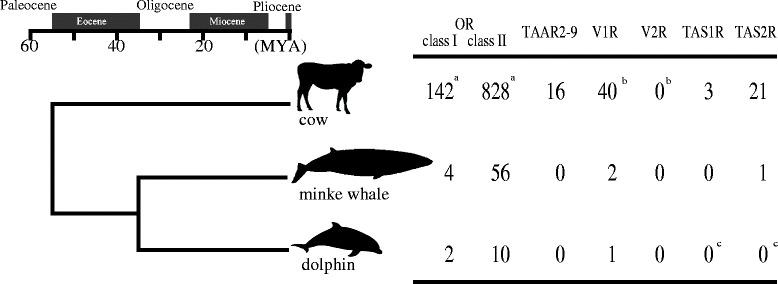
**Phylogenetic relationships, divergence times and the numbers of intact chemosensory receptor genes of cow (Artiodactyla), Antarctic minke whale (Mysticeti) and bottlenose dolphin (Odontoceti).** Notes: a. taken from Niimura and Nei [25]; b. taken from Shi and Zhang [52]; c. taken from Jiang *et al*. [7].

As Niimura and Nei pointed out [30], mammalian OR genes can clearly be classified into class I and class II based on the sequence similarity. Classification of OR genes into class I and II follows Glusman *et al.* [31] and Niimura and Nei [25], and the whale and dolphin intact OR genes identified in this study were classified into class I or II based on a phylogenetic tree which consists of deduced amino acid sequences of human (retrieved from HORDE database (http://genome.weizmann.ac.il/horde/) #43), cow, whale and dolphin intact *OR* genes (the phylogenetic tree is shown in Additional file 1 §3). In addition to 60 (minke whale) and twelve (dolphin) intact OR genes (Figure 2), we found 19 (minke whale) and two (dolphin) truncated OR genes. We added these truncated genes one by one to the OR phylogenetic tree and confirmed that all these truncated OR genes are classified into class II.

### Genes for the sense of taste

TBLASTN searches with e-value cutoff of <1e-5 and without filtering query sequences were employed to identify *TAS1R*, *TAS2R* and *GNAT3* genes. The amino acid sequence of cow GNAT3 is retrieved from GenBank (accession no. NP_001103452). Using all amniote GNAT3 sequences annotated in Ensembl database (http://www.ensembl.org/index.html) (release 73) as queries, *GNAT3* genes were searched against KUjira_1.0 and Ttru_1.4 assemblies. *TAS1R* genes were also searched against UMD_3.1, KUjira_1.0 and Ttru_1.4 assemblies using all vertebrate *TAS1R* sequences annotated in Ensembl database (release 70) as queries. In the case of TAS2Rs, we used all intact Euarchontoglires TAS2Rs identified by Hayakawa et al. [32] as queries and searched against UMD_3.1, KUjira_1.0 and Ttru_1.4 assemblies. All overlapping sequences of hits with the same orientations were merged. The sequences thus obtained were searched against the human (GRCh37 assembly) [33,34] and the mouse (GRCm38 assembly) [35] genome assemblies using TBLASTX and the sequence was discarded if its best hit was not a *GNAT3*/*TAS1R*/*TAS2R* gene. Because *TAS1R*s and *GNAT3* are multi-exon genes, the results of TBLASTX were also utilized for subsequent exon annotations. Exon regions and splicing sites of the *GNAT3* and *TAS1R* genes identified in this study were determined by comparing *GNAT3* and *TAS1R* sequences of cetartiodactyls with that of humans and mice using E-INS-i program in the MAFFT package. A taste receptor gene was considered a pseudogene or truncated gene if the same criteria were met that we followed for odorant receptors.

Gene annotation information thus obtained is available as Additional files 2, 3 and 4.

The 6^th^ exon of *GNAT3* genes in several cetaceans and artiodactyls (Additional file 1: Table S7) were sequenced using a pair of primers shown in Table 1.

**Table 1:** **Primers used for amplifying and sequencing the 6** ^**th**^ **exon of** ***GNAT3*** **gene**

**Name**	**Sequence**	**loci**
G3-6F	AGGTGGACAGAGATCTGARAG	Within 6th exon of GNAT3 gene
G3-6R	TATAAAAGATGAAAATGTGTAGGAT	Within 6th exon of GNAT3 gene
note:	This pair of primers are designed to amplify a 299 bp-length region including the partial amino coding region of the 6^th^ exon of *GNAT3* gene	

### Fossil investigation

**Figure 3:**
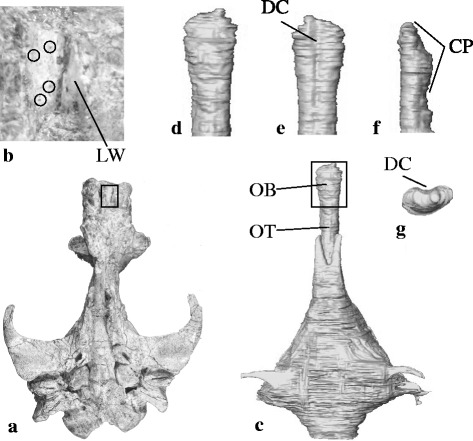
**Cribriform plate and olfactory bulb in extinct cetaceans.** **a.** Skull of the pakicetid cetacean *Ichthyolestes pinfoldi* (H-GSP 98134, described by Nummela et al. [36]) in ventral view, rectangle indicates detail shown in **(b)**. **b.** Detail of **(a)**, showing the dorsal side of the cribriform plate with some of its perforations encircled, and the lateral wall (LW) of the olfactory chamber. **c**. ventral view of the endocast of the cranial cavity of the remingtonocetid cetacean *Remingtonocetus* (IITR-SB 2770, described by Bajpai et al. [37]) based on 3D reconstruction of CT-scans, showing impressions of olfactory tract (OT) and olfactory bulb (OB), area in box is enlarged in **(d)**. **d**-**g**. impression of olfactory bulb in ventral, dorsal, lateral, and cranial view respectively, dorsal and rostral view show midline dorsal crest (DC), and lateral view shows the contrast between convex ventral side where cranial nerve I pierces the cribriform plate (CP), and flat dorsal side.

Several fossil whale skulls were used in this study. A skull of the pakicetid cetacean *Ichthyolestes pinfoldi* (Howard - Geological Survey of Pakistan, H-GSP 98134) was described by Nummela et al. [36]. Further preparation of sediment from the olfactory region of this specimen using an airscribe and dental tools, revealed the cribriform plate of this specimen. A specimen of the remingtonocetid cetacean *Remingtonocetus* (Indian Institute of Technology, Roorkee, IITR-SB 2770) was described by Bajpai et al. [37], and this specimen was CT-scanned, and 3D reconstructed using AMIRA software (FEI Visualization Science Group) ver. 5.4.1 as described by Bajpai et al. [37]. 3D reconstructions presented in Figure 3 were produced by AMIRA.

## Results

### Loss of the D domain in all modern whales

The D domain is defined by the expression of the *OMACS* gene [16,38], and the unique expression of *NQO1* gene is also reported [39]. We found that, in both minke whales and dolphins, the *OMACS* gene is not functional due to the loss of the 5^th^, 9^th^, 10^th^ and 11^th^ exons (Figure 1a). Both cetaceans also lost the functional *NQO1* gene due to genomic inversion (Figure 1b). These findings suggest that both *OMACS* and *NQO1* genes turned into pseudogenes before the Odontoceti-Mysticeti split.

The V domain is defined by the expression of the *OCAM* gene [16,40]. Minke whales have maintained the complete coding region of the *OCAM* gene. In addition to that, dolphins, even though they are anosmic, also have kept this gene under strict purifying selection (Additional file 1 §2), suggesting that this gene has unknown function besides olfaction.

The molecular basis of olfaction relies on the repertoires of four families of chemosensory receptors: TAAR, OR, V1R and V2R [1]. OSNs expressing TAARs project specifically to the D domain of the OB [41,42], and all of the *TAAR* genes are located in a single gene cluster with no interspersed genes [42]. We found that minke whales have lost the *TAAR5*, *6*, *7* and *8* genes and dolphins have lost the *TAAR2-8* genes. In addition, all of the remaining *TAAR* genes of whales and dolphins are functionless pseudogenes except for the whale *TAAR1* gene (Figure 1c), which is not involved in olfaction [43]. Deletion of TAAR5-8 genes from the minke whale genome was also confirmed by PCR (Additional file 1 §4). This finding suggests that both minke whales and dolphins have lost all the olfactory TAARs.

Mammalian ORs can be classified into two subfamilies, class I and class II, based on sequence similarity [30]. Most OSNs expressing class I ORs project specifically to the D domain [44], whereas OSNs expressing class II ORs project to both the D and V domains [45]. We found only four intact class I *OR* genes in the minke whale genome and two in the dolphin genome (Figure 2). Both whales and dolphins have kept two intact class I ORs, OR51E1 and OR51E2 (Additional file 1 §3). The expression of these two ORs is highly restricted to prostate tissues [46,47], indicating that these ORs play roles that are not related to olfaction. Minke whales have kept two more intact class I *OR* genes, but it is difficult to judge whether these two remaining genes are still functional or not. In any case, all cetaceans underwent a significant loss of olfactory-functional class I ORs in evolution. In contrast, 56 intact class II *OR* genes were found in the minke whale genome, well below the 828 present in cow [25], but above the ten in dolphins (Figure 2). This is consistent with our previous findings that V domain of the baleen whale OB is small but functional and that baleen whales have a sense of smell [10].

All of these findings suggest that mysticetes have lost most of the D domain-specific markers and receptors. We conclude that the mysticete OB lacks a region homologous to the D domain of the mouse OB. Putting the loss of the D domain markers in evolutionary perspective, we hypothesize that whales lost the D domain of the OB during the Eocene epoch, which is the time when whale ancestors migrated from land to water.

The OB communicates with the nasal cavity via the cribriform plate. The cribriform plate fossilizes and its shape can be used to deduce the shape of the OB, thus tracing the reduction of the D domain in evolutionary time. Cetaceans originated around 50 million years ago (MYA) [48], and their basal family is Pakicetidae [49]. In this family, the orbits are close together near the midline and the OB is located just anterior to the orbit [36]. As a consequence, the OB is very small [50]. However, we investigated the skull of a pakicetid *Ichthyolestes* and found that a part of the cribriform plate faces dorsally, and is perforated by many small foramina, presumably for cranial nerve I (Figure 3ab). On the other hand, the cribriform plate of remingtonocetids, Eocene whales closer to the divergence of mysticetes and odontocetes, differs from that of pakicetids, in that the OB faces ventral, similar to bowhead whales and there is no indication for dorsally projecting fibers of cranial nerve I (Figure 3c-g). Thus, whereas pakicetids show connections of the cribriform plate on the dorsal side of the OB, these connections are lost in remingtonocetids. In effect, the olfactory anatomy of modern minke whales resembles that of late Eocene whales [51]. This suggests that the D domain was lost during the course of the Eocene, but was present in the earliest cetaceans.

### Loss of the vomeronasal olfaction in basal cetaceans

No intact *V2R* genes exist in the cetaceans and cattle (Figure 2), suggesting that this gene family was lost in the cetartiodactyl lineage before the cow-cetacean split, congruent with a previous report [52]. In contrast, cattle have 40 intact *V1R* genes whereas mysticetes have only two and odontocetes just one (Figure 2). Absence of the VO, in which *V1R*s are expressed [1,53], can be inferred from fossils, and this suggests that the organ was lost around 45 MYA, before the divergence of odontocetes and mysticetes. The vomeronasal ducts of mammals pass through the anterior palatine foramina, and the absence of these foramina implies that the organ is absent. Whereas these foramina are still present in the earliest whales pakicetids [36], they have been lost in remingtonocetids [37], and it is likely that the VO was lost at this node of the cladogram.

### Loss of the sense of sweet, umami and bitter tastes in whales

**Figure 4:**
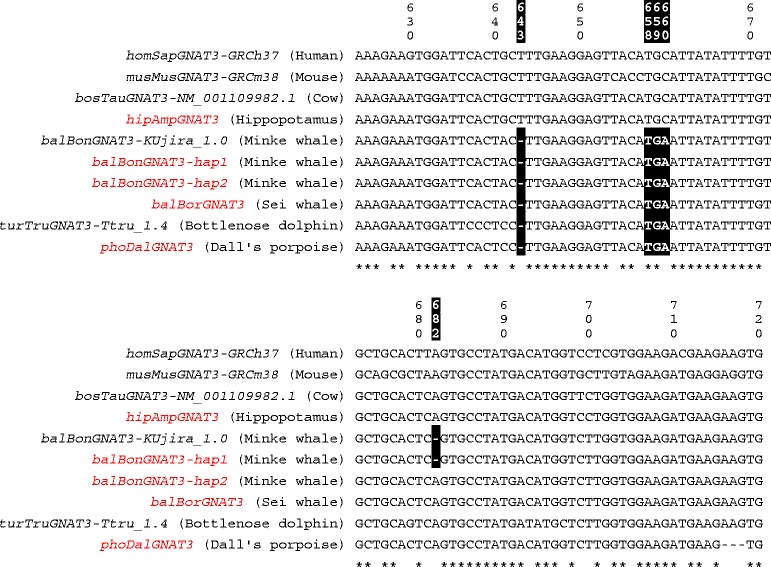
**A multiple alignment of partial nucleotide sequences of the 6th exon of** ***GNAT3*** **.** Human, mouse and cow *GNAT3* sequences were shown in addition to that of cetaceans and artiodactyls sequenced (shown with red fonts) or annotated in this study. Numbers above the alignment are corresponding to the nucleotide positions in the human *GNAT3*. Sequences based on Sanger-sequencing technology are indicated by red fonts. Asterisks under the alignment indicate non-variable nucleotide positions. Disrupting mutations and their positions are shaded. ‘Hap1’ and ‘hap2’ indicate haplotypes. All cetaceans share two lineage-specific pseudogenization mutations (a 1-bp deletion in the position 643 and a premature stop codon in the position 658–660).

Regarding taste receptors, no intact genes had not been found in cetacean genomes except for *TAS2R16* gene in common minke whale genome [7,8,13], and Feng et al. [12] suggested that several mysticetes possess an intact *TAS2R16* gene based on sequencing of partial coding regions. We confirmed that Antarctic minke whales have also lost all functional *TAS1R* and *TAS2R* genes except for one *TAS2R* gene, *TAS2R67*. We sequenced complete coding regions of *TAS2R16* and *TAS2R67* genes in several cetartiodactyl species and confirmed that the last common ancestor of odontocetes and mysticetes possessed intact *TAS2R16* and *TAS2R67* sequences (Additional file 1 §5). In addition, we found that the last common ancestors of odontocetes and mysticetes possessed an intact *TAS1R2* gene among *TAS1R* genes (Additional file 1 §5). However, proper function of the TAS1Rs and TAS2Rs requires their interaction with gustducin, and the GNAT3 (gustducin α-subunit)-KO mice show highly reduced responses to sweet and bitter taste [54]. Mammalian *GNAT3* gene consists of eight exons, and all exons of *GNAT3* except for the 2nd and 3rd exons were found in both KUjira_1.0 and Ttru_1.4 assemblies. Several pseudogenization mutations (frame shift mutations and premature stop codons) were found in both the whale and dolphin *GNAT3* sequences, indicating that both whales and dolphins lost the functional GNAT3. Especially among such mutations, whales and dolphins share two pseudogenization mutations (1 bp deletion and a premature stop codon) in the 6th exon. We sequenced 6th exon of the *GNAT3* gene and found that all cetaceans have lost the GNAT3, by means of a frame shift mutation and a premature stop codon in the 6^th^ exon (Figure 4). These findings suggest that the gustatory capability had been greatly reduced in cetaceans between the Artiodactyla-Cetacea split and the Odontoceti-Mysticeti split.

## Discussion

Taken together, these results describe the outline of chemosensory evolution in cetaceans during the land to water transition. Cetaceans are derived from artiodactyls with well-developed olfactory and vomeronasal organs [55,56], although their V2Rs were already lost [52]. Amphibious basal cetaceans emerged around 50 MYA, when olfactory organs were reduced, but retained both D and V domains. Around 45 MYA, the cetacean family Remingtonocetidae underwent significant changes in their chemical senses, losing the VO and the D domain of the OB. At this time, *V1R*s, *OMACS*, *NQO1*, olfactory *TAAR*s as well as most of class I *OR* genes are speculated to have lost their functions. *Remingtonocetus* are considered to have been one of the earliest whales that acquired well-established underwater hearing systems [37], and it is possible that, at this point in evolution, the importance of olfaction as a sense decreased. Basically, these conditions were maintained in modern mysticete whales. It is not obvious that physiological studies using mice can be directly extended to other mammals. However, it is reasonable to assume that the olfactory capability of mysticetes is similar to that of ΔD mice, i.e., that mysticetes lack innate avoidance behavior against predator odors and spoiled smells. Terrestrial animals cannot prey on fully aquatic whales, and whales’ predators, such as sharks and killer whales, cannot be detected by smelling in air. In addition, unlike the nares of other mammals, whales’ nares are not located at the tip of their snout, and whales’ nasal passage is not connected directly to their oral cavity, indicating that it is difficult for whales to rely on olfaction to judge whether something they are about to swallow is edible or not. Further studies will test this assumption. The evolution of taste cannot be traced in mysticete evolution, but gene evidence indicates that all cetaceans lack functional receptors for sweet, bitter and umami flavors. Although some Neogene odontocetes had a large OB chamber and well-developed cribriform plate [57], modern odontocetes reduced their chemical senses even further, losing the entire OB with further loss of class II *OR* genes.

This study indicates that all modern cetaceans lack innate avoidance behavior against spoiled smells, and the sense of tastes. This could be one of the reasons why cetaceans often die from ingesting inedible debris [58], and has implications for whale conservation.

## Conclusion

**Figure 5:**
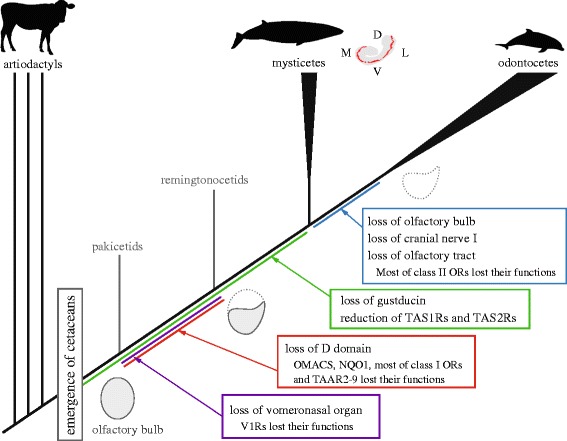
**Simplified phylogeny of cetaceans discussed here with evolutionary events indicated.** Distribution pattern of the glomeruli on the coronal section of OB of a modern mysticete (modified from Thewissen et al. [10]; D, dorsal; M, medial; V, ventral; L, lateral) , and evolutionary changes of the speculated shapes of the coronal section of OB, are also shown. Our findings indicate that profound changes on olfactory systems occurred just before the modern whales-remingtonocetids split. *Remingtonocetus* are suggested to have been ambush predators that were one of the earliest whales living in seawater [37]. They had well-established capabilities of underwater hearing, but their ecology remains largely elusive.

We showed that, though mysticetes possess functional olfactory bulbs, they had lost the D domain of the olfactory bulb during the Eocene Epoch. In addition, all modern whales have lost the functional sweet, umami and bitter taste receptors before the Odontoceti-Mysticeti split (Figure 5). These findings suggest that profound changes in the chemosensory capabilities occurred in the cetacean lineage before the Odontoceti-Mysticeti split, during the period when ancestral whales migrated from land to water.

### Availability of supporting data

The Antarctic minke whale genome data sets supporting the results of this article are available in the DDBJ/EBI/NCBI databases under the following BioProject ID: PRJDB1465. The 6^th^ exons of the *GNAT3* genes amplified and sequenced in this study are available in the DDBJ/EMBL/GenBank databased under the following accession numbers: AB897678, AB897679, AB897680, AB897682 and AB897683.

## Additional files

Additional file 1:
**This additional file provides the supplementary information on 1) Antarctic minke whale (Balaenoptera bonaerensis) genome assembly; 2) Evolution of** ***OCAM*** **gene in the cetacean lineage; 3) Phylogenetic analyses and classification of** ***TAAR*** **,** ***OR*** **and** ***V1R*** **genes; 4) Amplification of a genomic region between** ***TAAR4*** **and** ***TAAR9*** **genes in the minke whale genome; and 5) Evolution of the taste receptor genes in cetaceans.**
Additional file 2:
**Gene annotation on** ***Balaenoptera bonaerensis*** **genome assembly (KUjira_1.0).**
Additional file 3:
**Gene annotation on** ***Tursiops truncatus*** **genome assembly (Ttru_1.4).**
Additional file 4:
**Gene annotation on** ***Bos taurus*** **genome assembly (UMD_3.1).**

## Abbreviations

D domain: Dorsal domain
GNAT3: Guanine nucleotide binding protein, alpha transducing 3
GPCR: G-protein coupled receptor
MYA: Million years ago
NQO1: NAD(P)H dehydrogenase quinone 1
OB: Olfactory bulb
OCAM: Olfactory cell adhesion molecule
OMACS: Olfactory medium-chain acyl-CoA synthetase
OR: Olfactory receptor
OSN: Olfactory sensory neuron
TAAR: Trace amine-associated receptor
TAS1R: Taste receptor type 1
TAS2R: Taste receptor type 2
TM: Trans-membrane
V domain: Ventral domain
V1R: Vomeronasal receptor type 1
V2R: Vomeronasal receptor type 2
VO: Vomeronasal organ
