# Supplementary material for: Aquatic adaptation and the evolution of smell and taste in whales
Source: Zoological Lett. 2015 Feb 13;1:9. doi: 10.1186/s40851-014-0002-z (PMC4604112; doi:10.1186/s40851-014-0002-z)
Supplement: Additional file 1: — This additional file provides the supplementary information on 1) Antarctic minke whale (Balaenoptera bonaerensis) genome assembly; 2) Evolution of OCAM gene in the cetacean lineage; 3) Phylogenetic analyses and classification of TAAR , OR and V1R genes; 4) Amplification of a genomic region between TAAR4 and TAAR9 genes in the minke whale genome; and 5) Evolution of the taste receptor genes in cetaceans. [file 40851_2014_2_MOESM1_ESM.pdf]

Aquatic adaptation and the evolution of smell and taste in whales

Takushi Kishida, J. G. M. Thewissen, Takashi Hayakawa, Hiroo Imai, Kiyokazu Agata

Supplementary Information

1. Antarctic minke whale (*Balaenoptera bonaerensis*) genome assembly

2. Evolution of *OCAM* gene in the cetacean lineage

3. Phylogenetic analyses and classification of *TAAR*, *OR* and *V1R* genes

4. Amplification of a genomic region between *TAAR4* and *TAAR9* genes in the minke whale genome

5. Evolution of the taste receptor genes in cetaceans

1. Antarctic minke whale (*Balaenoptera bonaerensis*) genome assembly

1.1 Genome sequencing

We used a whole genome shotgun strategy and the next-generation sequencing technologies on the Illumina HiSeq 2000 sequencer to sequence the genome of Antarctic minke whale *Balaenoptera bonaerensis*. Whale DNA was extracted from muscle tissue of an individual, that was purchased from a fish market in Japan. Paired-end TruSeq sequencing library with average insert size of 330bp was constructed and sequenced (100bp x2, eight lanes in total). As a result, 1.43G read-pairs, 285Gbp were obtained (Table S1).

1.2 Trimming low-quality regions and adapter sequences

Trimmomatic[1] ver. 0.30 was used to trim low-quality regions and adapter sequences. Parameters were as follows: ILLUMINACLIP:Trimmomatic-0.30/adapters/TruSeq3-PE.fa:2:30:10 LEADING:20 TRAILING:20 SLIDINGWINDOW:20:30 MINLEN:36. The results are shown in Table S1.

1.3 Genome-size estimation and sequence-error correction based on the *k-mer* frequency spectrum

KmerFreq_HA program in the SOAPec ver. 2.01 package[2] was used to construct the *k-mer* frequency spectrum (*k=21*) (Fig. S1). Following Fig. S1, the total number of *k-mer*s (*K_num*) was 195,248,671,032 and the peak depth (*K_depth*) was 60, respectively. Assuming that the genome size *G* can be estimated as *G=K_num/K_depth*, the Antarctic minke whale genome size was estimated to be 3.25Gbp. Note that this genome size would be overestimated because the clearly confirmable heterozygous peak (Fig. S1) increases the *K_num* value.

Assuming that most low-frequency *k-mer*s were generated by sequencing errors, Corrector_HA program in the SOAPec package was used to correct these errors. Parameters were as follows: -k 21 -j 1 -l 6 -e 1 -w 1 -q 30 -r 36. As a result, 249Gbp sequences, as shown in Table S1, were retained for genome assembly.

Table S1. Numbers of reads and bases of the Antarctic minke whale genome sequences

	paired-end reads		unpaired reads		total	
	no. of reads	no. of bases	no. of reads	no. of bases	no. of reads	no. of bases
HiSeq2000 output	1.4273G x2	285.46G	0	0	2.8546G	285.46G
after Trimmomatic	1.2587G x2	241.31G	116.72M	8.3858G	2.6342G	249.69G
after SOAPec	1.2576G x2	240.90G	117.33M	8.4348G	2.6336G	249.33G

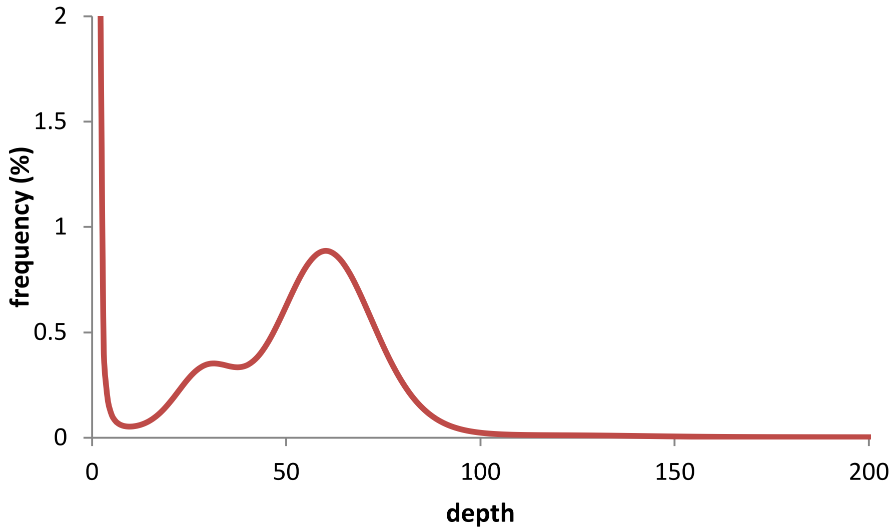

Figure S1. *k-mer* frequency spectrum (*k=21*)

1.4 Genome assembly

As shown in Fig. S1, the Antarctic minke whale genome is highly heterozygous. Therefore, PLATANUS [3] ver. 1.2.1 assembler (http://platanus.bio.titech.ac.jp/platanus-assembler/) was employed for *de novo* assembling, scaffolding and gap-closing because it can handle highly heterozygous data.

The command-lines inputted for genome assembly were as follows;

$ platanus assemble -t 20 -f [sequence files] -u 0.2 -s 4 -d 0.3 -m 256

$ platanus scaffold -c [contig file] -b [contig bubble file] -IP [PE sequence files] -t 20 -u 0.2

$ platanus gap_close -c [scaffold file] -IP [PE sequence files] -t 20

Statistics of assembled sequences are shown in Table S2. Total length of the contig assembly is as long as the estimated genome size (see §1.3). However, the total length of the scaffold assembly is much lower than that. It can be explained that PLATANUS assembler clashed bubbles which originated in heterozygous alleles efficiently. Actually, a common minke whale genome assembly was reported when we wrote this paper, and the total length of scaffolds of the common minke whale was 2.44Gbp[4], consisting with our results.

The *Balaenoptera bonaerensis* genome assembly thus obtained was named KUjira_1.0.

The sequenced reads and assembled genome are available in the DDBJ/EBI/NCBI databases under the following BioProject ID: PRJDB1465. Accession no. of the sequenced short reads is DRR014695. Accession nos. of assembled scaffolds are DF397027-DF818470.

Table S2. Statistics of assembled sequences

	contig	scaffold	gap-closed
no. of sequences	13,383,615	421,444	421,444
total length (bp)	3,279,645,845	2,236,396,878	2,234,641,557
N25 (bp)	4,624	34,096	34,073
N50 (bp)	1,740	20,093	20,082
N75 (bp)	127	10,520	10,513
longest sequence (bp)	43,414	189,786	189,744
GC content (%)	41.58	40.16	40.48
rate of 'N' (%)	0	1.35	0.64

2. Evolution of *OCAM* gene in the cetacean lineage

The V domain of the OB is characterized by the expression of the *OCAM* gene. We found that both minke whale and dolphin have kept an intact *OCAM* gene in their genomes. To understand the presence or absence of purifying selection on this gene in the anosmic odontocetes lineage, we analyzed the nonsynonymous to synonymous substitution rate ratio ω (d_n/d_s) in cetacean branches based on the maximum likelihood (ML) method. The *OCAM* genes of two artiodactyls, cow (GenBank accession no. XM_002684635.2) and sheep (XM_004002813.1) were used as outgroups. DNA sequences of whale, dolphin, cow and sheep *OCAM* genes were aligned using the L-INS-i program in the MAFFT package[5, 6] ver. 6.240 with manual adjustments, and the CODEML program in the PAML4.4 package[7] was used to analyze changes of selective pressure on each branch (Fig. S2). In this analysis, the transition/transversion rates were not fixed and the F3×4 model was used for codon usage biases.

The ω ratio on the dolphin branch is as small as that on the other branches. To test whether this gene has been evolved under the presence of purifying selection on this branch or not, we followed the method of Zhang *et al.*[8] Following the method of Nei and Gojobori[9], the numbers of nonsynonymous (N) and synonymous (S) sites of the dolphin OCAM gene were 1615 and 470, respectively. As shown in Table S3, this gene has significantly ($p<0.01$) evolved under the presence of strict purifying selection even on the 'anosmic' dolphin branch.

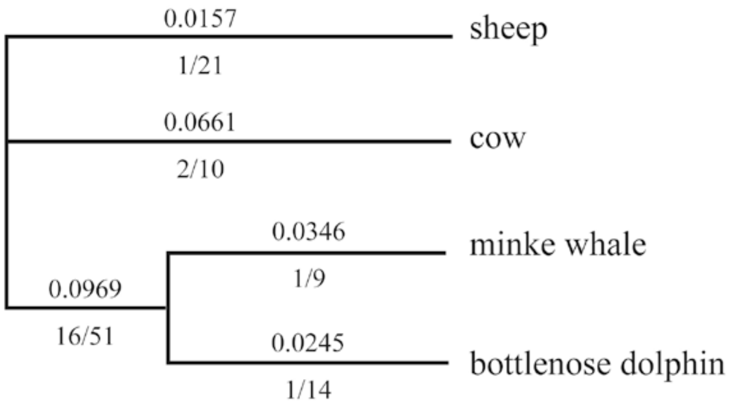

Figure S2. The nonsynonymous to synonymous rate ratios (ω) in each branch, calculated based on the free-ratio model. The estimated numbers of nonsynonymous substitutions/synonymous substitutions, calculated by the method of Nei and Gojobori[9], are also shown under each branch based on the ancestral nucleotide sequences inferred by the Bayesian method[10].

Table S3. Test of purifying selection on the dolphin branch

	Nonsynonymous	Synonymous	Prob.*
Changes	1 (n)	14 (s)	
No changes	1614 (N-n)	456 (S-s)	8.96×10^{-9}

Note: * tail probability in Fisher's exact test of homogeneity.

3. Phylogenetic analyses and classification of *TAAR*, *OR* and *V1R* genes

In order to classify cow *TAAR* genes into *TAAR1-9* and cetacean *OR* genes into class I/II, and to find whale-dolphin orthologous genes, phylogenetic trees of deduced amino acid sequences of intact *TAAR*, *OR* and *V1R* genes were inferred. Trees were constructed by the neighbor-joining (NJ) method[11], based on the Poisson correction distance matrices. Sequences were aligned using the L-INS-i program, and gap sites were excluded from the analyses. Bootstrap values were obtained by 500 resamplings, and the calculations were carried out using MEGA5[12]. *TAAR1* genes were rooted in Fig. S3, as suggested by Hashiguchi and Nishida[13]. Class I ORs and class II ORs were rooted reciprocally in Fig. S4, as suggested by Niimura and Nei[14]. The V1R tree (Fig. S5) is unrooted. Seventeen cow intact *TAAR* genes were classified into TAAR1-9 as shown in Fig. S3. Whale and dolphin intact OR genes were classified into class I/II as shown in Fig. S4. Figure S4 shows that both minke whales and dolphins have maintained two class I *OR* genes, *OR51E2* and *OR51E1*. These genes are also known as PSGR (prostate-specific G-protein coupled receptor)[15] and PSGR2[16], respectively, and are known to express specifically in the prostate tissue. In addition to these two class I ORs, both whale and dolphins have maintained four class II intact ORs (OR6B1, OR6Q1, BotaORs802.8 and OR2AT4). Not only OR51E1 and OR51E2 but also these four class II OR orthologs might have functions unrelated to olfaction. Actually, OR6Q1 is known to express in epithelial cells on the surface of the tongue[17], suggesting that it has a non-olfactory function. In contrast, Fig. S5 shows that minke whales and dolphins do not share any orthologous *V1R* genes.

Genbank accession nos. of human and mouse *TAAR* genes used in Fig. S3 are shown in Fig. S3. Human intact ORs were retrieved from the HORDE database #43 (http://genome.weizmann.ac.il/horde/). Opossum, dog and cow intact V1R sequences were obtained from Grus *et al*.[18]

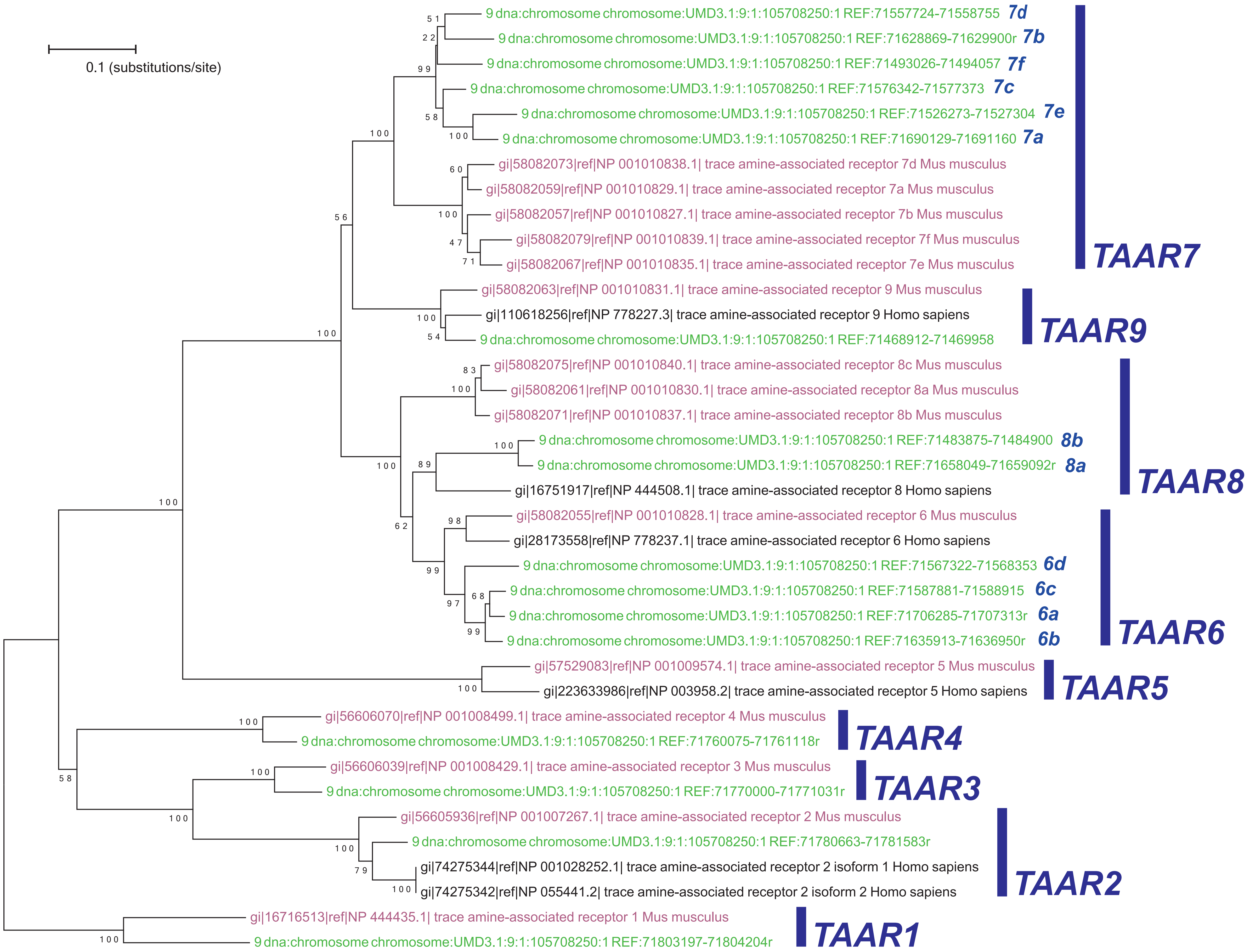

Figure S3. A NJ tree of deduced amino acid sequences of human (black), mouse (magenda) and cow (green) intact ***TAAR*** genes

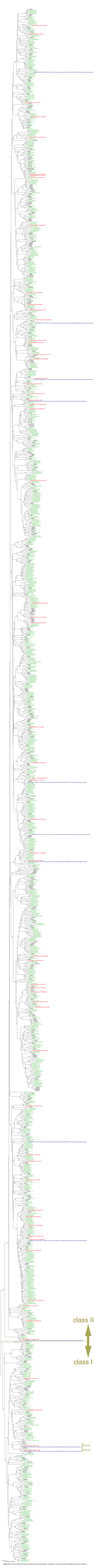

Figure S4. A NJ tree of deduced amino acid sequences of human (black), cow (green), minke whale (red) and dolphin (blue) intact OR genes

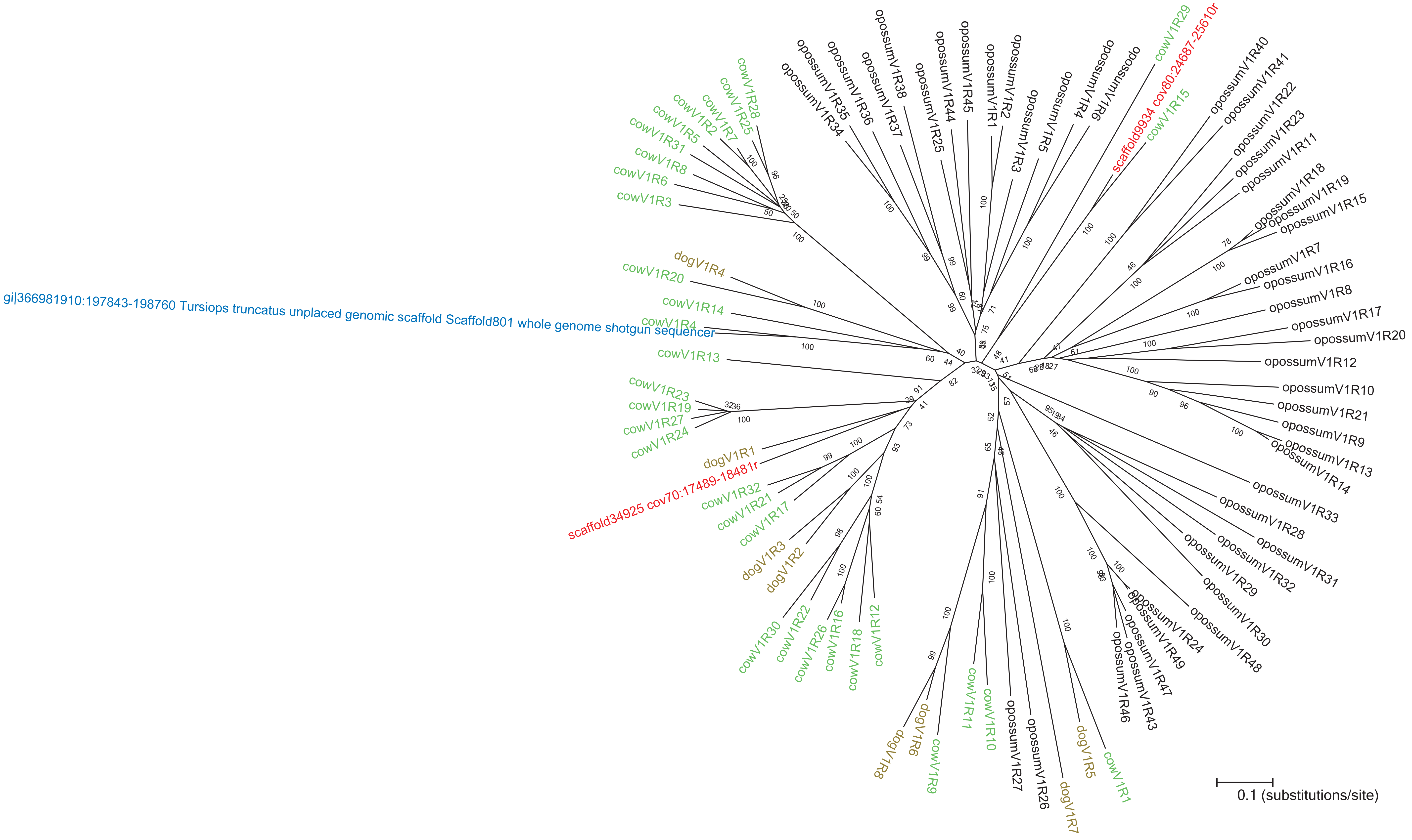

Figure S5. A NJ tree of deduced amino acid sequences of opossum (black), dog (light brown), cow (green), minke whale (red) and dolphin (blue) intact *V1R* genes

4. Amplification of a genomic region between *TAAR4* and *TAAR9* genes in the minke whale genome

As shown in Fig. 2c, a large-scale deletion was confirmed in the genomic region between *TAAR4* and *TAAR9* genes in the minke whale genome. However, unlike in the case of the *OMACS* (Fig. 2a) and *NQO1* (Fig. 2b) genes, minke whale does not share the same genomic deletion with dolphin, indicating that the accuracy of KUjira_1.0 assembly is not validated by comparing the assembly with that of related species. Therefore, we amplified the genomic region between the *TAAR4* and *TAAR9* genes of Antarctic minke whale genome using a pair of primers shown in Table S4. PCR amplification was performed using AccuPrime *Taq* DNA Polymerase High Fidelity (Invitrogen) with 30 cycles of denaturation (94°C for 20sec.), annealing (60°C for 20sec.) and extension (68°C for 15min.). The expected length of the PCR amplicon of minke whale calculated based on the KUjira_1.0 assembly was 54747-45891 = 8856bp. The actual length of the PCR amplicon of minke whale was equal to the expected length (Fig. S6), indicating that our whale genome assembly is accurate. We sequenced the both ends of this amplicon on an automated sequencer (ABI3130, Applied Biosystems) using TAAR9f and TAAR4f primers as sequencing primers and confirmed that we correctly amplified the target region.

Table S4. Primers used for *TAAR* gene cluster amplification

name	sequence	locus
TAAR9f	ACTTCTTGGTGGGAGTGACTGTGA	scaffold12993, 54724-54747bp (complement) (within *TAAR9* gene)
TAAR4f	TTCCATTGCCCACTTCAAGCAGCT	scaffold12993, 45892-45915bp (within *TAAR4* gene)

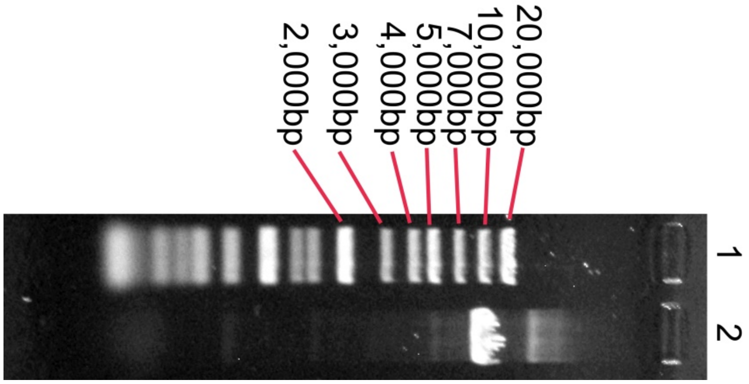

Figure S6. The length of PCR amplicons. Lane 1, DNA ladder (Gene Ladder Wide 2, Nippon Gene); lane 2, PCR amplicon of genomic DNA of Antarctic minke whale *Balaenoptera bonaerensis*, which was extracted in §1.1.

5. Evolution of the taste receptor genes in cetaceans

We annotated nucleotide sequences of the *TAS1R* (umami and sweet taste receptor) and *TAS2R* (bitter taste receptor) gene families in the minke whale, dolphin, and cow genome assemblies (see Supplementary Data 1-3).

In all the assemblies, the *TAS1R* family was composed of three genes, *TAS1R1*, *TAS1R2*, and *TAS1R3*. In the cow genome assembly, all exons of *TAS1R*s did not include local disrupting mutations. However, a contig containing the 4th, 5th, and 6th exons of *TAS1R3* was inversely located against a contig containing the 1st, 2nd, and 3rd exons in the chromosomal assembly. This may be due to misassembling because an alternative genome assembly of the cow (Btau_4.6 assembly, ftp://ftp.ncbi.nlm.nih.gov/genbank/genomes/Eukaryotes/vertebrates_mammals/Bos_taurus/Btau_4.6/) continuously located *TAS1R3* from 1st to 6th exons in 100% nucleotide sequence identity with *TAS1R3* of the investigated cow UMD_3.1 assembly. Therefore, the cow would have three intact *TAS1R*s. On the other hand, all the whale and dolphin *TAS1R*s are revealed to be pseudogenes because of disrupting mutations.

The multiple alignments of each *TAS1R* in the cow, minke whale and dolphin as well as the human and mouse revealed that minke whales and dolphins share same pseudogenization mutations in the *TAS1R1* gene and the *TAS1R3* gene but they do not share any pseudogenization mutations in the *TAS1R2* gene. Unfortunately, the 4th exon of *TAS1R2*, where a frame-shift mutation was confirmed in whale KUjira_1.0 assembly, was not found from the dolphin Ttru_1.4 assembly. In order to reveal whether mysticetes and odontocetes share the same frame-shift mutation in the 4th exon of *TAS1R2* gene or not, we amplified and sequenced the whole 4th exon of *TAS1R2* in an odontocete genome as well as three mysticete genomes (see Table S7) using a pair of primers shown in Table S5. The 4th exon of *TAS1R2* was not disrupted in all cetaceans except for that in minke whales (Fig. S7). Considering all these things, we conclude that the*TAS1R1* and *TAS1R3* genes were turned into pseudogenes before the Mysticeti-Odontoceti split, but the last common ancestor (LCA) of mysticetes and odontocetes possessed intact *TAS1R2* gene.

Table S5. Primers used for amplifying and sequencing the 4th exon of *TAS1R2*gene

name	sequence	loci
2-4F	CTCCAAACTGCCACATTTT	between the 3rd and 4th exons of theTAS1R2 gene
2-4R	CCAGACAGTACACCCACAAA	between the 4th and 5th exons of theTAS1R2 gene

note: This pair of primers are designed to amplify a 467bp-length region including the whole amino coding region of the 4th exon of *TAS1R2* gene

```
                                        1         1         1         1         1         1
                                        2         2         2         2         3         3
                                        6         7         8         9         0         1
                                        0         0         0         0         0         0
          homSapTAS1R2-GRCh37 (Human) CTGCTTGAGGAGATCTGGAAGGTCAACTTCACTCTCCTGGACCACCAAATCTTCTTCGAC
          musMusTAS1R2-GRCm38 (Mouse) CTACTCAGGGAGATCTGGCATGTCAACTTCACGCTCCTGGGCAACCAGCTCTTCTTCGAC
           bosTauTAS1R2-UMD_3.1 (Cow) CTGCTTCTGGAGATCAGGAAGGTCAACTTCACCCTCCTGGGCCACCAAATCTTCTTCAAC
balBonTAS1R2-KUjira_1.0 (Minke whale) CTACTTCTGGAGATCTAGAAGGTCAACTTCACCCTCCTGGGCCACCAAATCTTCTTCAAT
           balBonTAS1R2 (Minke whale) CTACTTCTGGATATCTGGAAGGTCAACTTCACCCTCCTGGGCCACCAAATCTTCTTCAAT
             balBorTAS1R2 (Sei whale) CTACTTCCGGAGATCTGGAAGGTCAACTTCACCCTCCTGGGCCACCAAATCTTCTTCAAT
kogSimTAS1R2-hap1 (Dwarf sperm whale) CTACTTCCGGAGATCTGGAAGGTCATCTTCACCCTCCTGGGCCACCCGA---TCTTCAGT
kogSimTAS1R2-hap2 (Dwarf sperm whale) CTACTTCCGGAGATCTGGAAGGTCATCTTCACCCTCCTGGGCCACCCGA---TCTTCAGT
                                      ** **   *** ***  * * **** ****** ******* * ***      *****

                                        1         1         1         1         1         1
                                        3         3         3         3         3         3
                                        2         3         4         5         6         7
                                        0         0         0         0         0         0
          homSapTAS1R2-GRCh37 (Human) CCGCAAGGGGACGTGGCTCTGCACTTGGAGATTGTCCAGTGGCAATGGGACCGGAGCCAG
          musMusTAS1R2-GRCm38 (Mouse) GAACAAGGGGACATGCCGATGCTCCTGGACATCATCCAGTGGCAATGGGGCCTGAGCCAG
           bosTauTAS1R2-UMD_3.1 (Cow) GAGCAAGGGGACCTGTCCCTGAACCTGGAGATCATCCAGTGGCAATGGGACCTGAGCCAG
balBonTAS1R2-KUjira_1.0 (Minke whale) GAGCAAGGGGACCTGTCCCTGCACCTGGAGGTCATCCAGTGGCAATGGCACCTGAGTCAG
           balBonTAS1R2 (Minke whale) GAGCAAGGGGACCTGTCCCTGCACCTGGAGGTCATCCAGTGGCAATGGCACCTGAGTCAG
             balBorTAS1R2 (Sei whale) GAGCAAGGGGACCTGTCCCTGAACCTGGAGGTCATCCAGTGGCAATGGGACCTGAGCCAG
kogSimTAS1R2-hap1 (Dwarf sperm whale) GAGCAAGGGGACCTTCCCCTGCACCTGGAGGTCATCCAGTGGCAATGGGACCTGAGGCAG
kogSimTAS1R2-hap2 (Dwarf sperm whale) GAGCAAGGGGACCTTCCCCTGCACCTGGAGGTCATCCAGTGGCAATGGGACCTGAGCCAG
                                         ********* *  *  ** * ****  *  **************  ** *** ***

                                      1 1         1         1         1         1         1
                                      3 3         3         4         4         4         4
                                      7 8         9         0         1         2         3
                                      8 0         0         0         0         0         0
          homSapTAS1R2-GRCh37 (Human) AATCCCTTCCAGAGCGTCGCCTCCTACTACCCCCTGCAGCGACAGCTGAAGAACATCCAA
          musMusTAS1R2-GRCm38 (Mouse) AACCCCTTCCAAAGCATCGCCTCCTACTCCCCCACCGAGACGAGGCTGACCTACATTAGC
           bosTauTAS1R2-UMD_3.1 (Cow) AACCCTTTTCGGAGCGTCGCCTCCTACCAGCCATCCAAGCGGCAGCTGAAGCACATCCAT
balBonTAS1R2-KUjira_1.0 (Minke whale) -ACCCTTTCCAGAGCATCGCCTCCTACCAACCGTCGAAGCGGCAGCTGAAGGACATTTGT
           balBonTAS1R2 (Minke whale) -ACCCTTTCCAGAGCATCGCCTCCTACCAACCGTCGAAGCGGCAGCTGAAGGACATTTGT
             balBorTAS1R2 (Sei whale) AACCCTTTCCAGAGCATCGCCTCCTACCAACCGTCGAAGCGGCAGCTGAAGGACATTTGT
kogSimTAS1R2-hap1 (Dwarf sperm whale) AGCCCTTTCCAGAGCATCGCCTCCTGCCAACCGTCGAAGTGGCAGCTGAAGGACGCTTGT
kogSimTAS1R2-hap2 (Dwarf sperm whale) AGCCCTTTCCAGAGCATCGCCTCCTGCCAACCATCGAAGTGGCAGCTGAAGGACGCTTGT
                                         ** ** *  *** ********* *   **     **     *****   **

                                        1         1         1
                                        4         4         4
                                        4         5         6
                                        0         0         0
          homSapTAS1R2-GRCh37 (Human) GACATCTCCTGGCACACCATCAACAACACG
          musMusTAS1R2-GRCm38 (Mouse) AATGTGTCCTGGTACACCCCCAACAACACG
           bosTauTAS1R2-UMD_3.1 (Cow) GACATCTCCTGGAACACCCCCAACAACACG
balBonTAS1R2-KUjira_1.0 (Minke whale) GACATCTCCTGGCACACCCCTAACAGCACG
           balBonTAS1R2 (Minke whale) GACATCTCCTGGCACACCCCTAACAGCACG
             balBorTAS1R2 (Sei whale) GACATCTCCTGGCACACCCCTAACAGGACG
kogSimTAS1R2-hap1 (Dwarf sperm whale) GACATCTCCCGGCACACTCCTAACAGCATG
kogSimTAS1R2-hap2 (Dwarf sperm whale) GACATCTCCCGGCACACTCCTAACAGCATG
                                       *  * *** ** ***     ****  * *
```

Figure S7. A multiple alignment of the nucleotide sequences of the complete 4th exon of *TAS1R2*. Numbers above the alignment are corresponding to the nucleotide positions in the human *TAS1R2*. Sequences based on Sanger-sequencing technology are indicated by red fonts. Asterisks under the alignment indicate non-variable nucleotide positions. 'hap1' and 'hap2' indicate haplotypes. Pseudogenization mutations were found only in the minke whales.

Concerning *TAS2R*s, 20 intact genes and one truncated gene (*TAS2R7*) were found in the cow UMD_3.1 assembly, whereas only an intact and a truncated genes (*TAS2R67* and *TAS2R2TA*, respectively) were found in the whale KUjira_1.0 assembly, and neither intact nor truncated genes were found in the dolphin Ttru_1.4 assembly. The truncated gene, *TAS2R2TA*, in the minke whale might be derived from the same loci of a pseudogene, *TAS2R2BP*, because *TAS2R2BP* was also located in the scaffold end and *TAS2R2TA* and *TAS2R2BP* were not overlapped each other in the seven-TM topology. There were 16, 12, and 11 pseudogenes in the cow, minke whale, and dolphin genome assemblies, respectively. A gene tree of all the *TAS2R* genes was shown in Fig. S8. The *TAS2R* gene repertoire in the dolphin Ttru_1.4 assembly is consistent with a previous study[19] except for *TAS2R62BP*, which is not annotated in the previous study.

We constructed the multiple alignment of each *TAS2R* orthologous gene set using E-INS-i program in the MAFFT package[5, 6] to understand when cetacean *TAS2R*s became pseudogenes. We found that both mysticetes and odontocetes share the same pseudogenization mutations in all sets of orthologous *TAS2R* genes except for *TAS1R16* and *TAS2R67*. Whales and dolphins do not share any pseudogenization mutations in the *TAS2R16* gene, and *TAS2R67* gene, which we could not find in the dolphin genome, is intact in the minke whale genome. This indicates that the LCA of mysticetes and odontocetes possessed at least 2 intact *TAS2R*s, *TAS2R16* and *TAS2R67*. We amplified and sequenced the *TAS2R16* and *TAS2R67* genes in several cetartiodactyls using several sets of primers shown in Table S6 to validate this indication (see Table S7). We confirmed that the *TAS2R16* gene has been pseudogenized independently in mysticetes and odontocetes (Fig. S9a). *TAS2R67* was not amplified in any odontocetes (Table S7). This gene may be deleted completely in all odontocetes. Not only minke whales but also sei whales possess intact *TAS2R67* gene. Hippopotamus lost this gene (Fig. S9b).

Table S6. Primers used for amplifying and sequencing *TAS2R16* and *TAS2R67* genes

name	sequence	target	usage	direction
16F	TGCATCCAGAGGGAGATATT	*TAS2R16*[a]	PCR and sequencing	F
16R	GATGGCTGTAGGGAAAGAGA	*TAS2R16*[a]	PCR and sequencing	R
16inF	GCTGTACAACTTTTGCTCCC	*TAS2R16*	sequencing	F
16inR	CAGGGCAGTAGAGTGAGCTT	*TAS2R16*	sequencing	R
67F	TGATGAGTGGGCACATTTAC	*TAS2R67*[b]	PCR and sequencing	F
67R	TGACAATGATGTTCGCTTTT	*TAS2R67*[b]	PCR and sequencing	R
67inF	GGGAATGGGTTCATTGTACT	*TAS2R67*[b]	PCR and sequencing	F
67inR	TGGCAGTTAAGATACCACAGTAG	*TAS2R67*[b]	PCR and sequencing	R

notes: [a]A pair of PCR primers 16F and 16R is designed based on Kujira_1.0 assembly to amplify a 1,171bp-length region including the whole amino acid coding region of *TAS2R16* gene.

[b]A pair of primers 67F and 67R is designed based on KUjira_1.0 assembly to amplify a 1,414bp-length region including the whole amino acid coding region of *TAS2R67* gene, and a pair of primers 67inF and 67inR is designed to amplify a partial sequence of the amino acid coding region of *TAS2R67* gene (833bp-length, used to amplify hippopotamus *TAS2R67*).

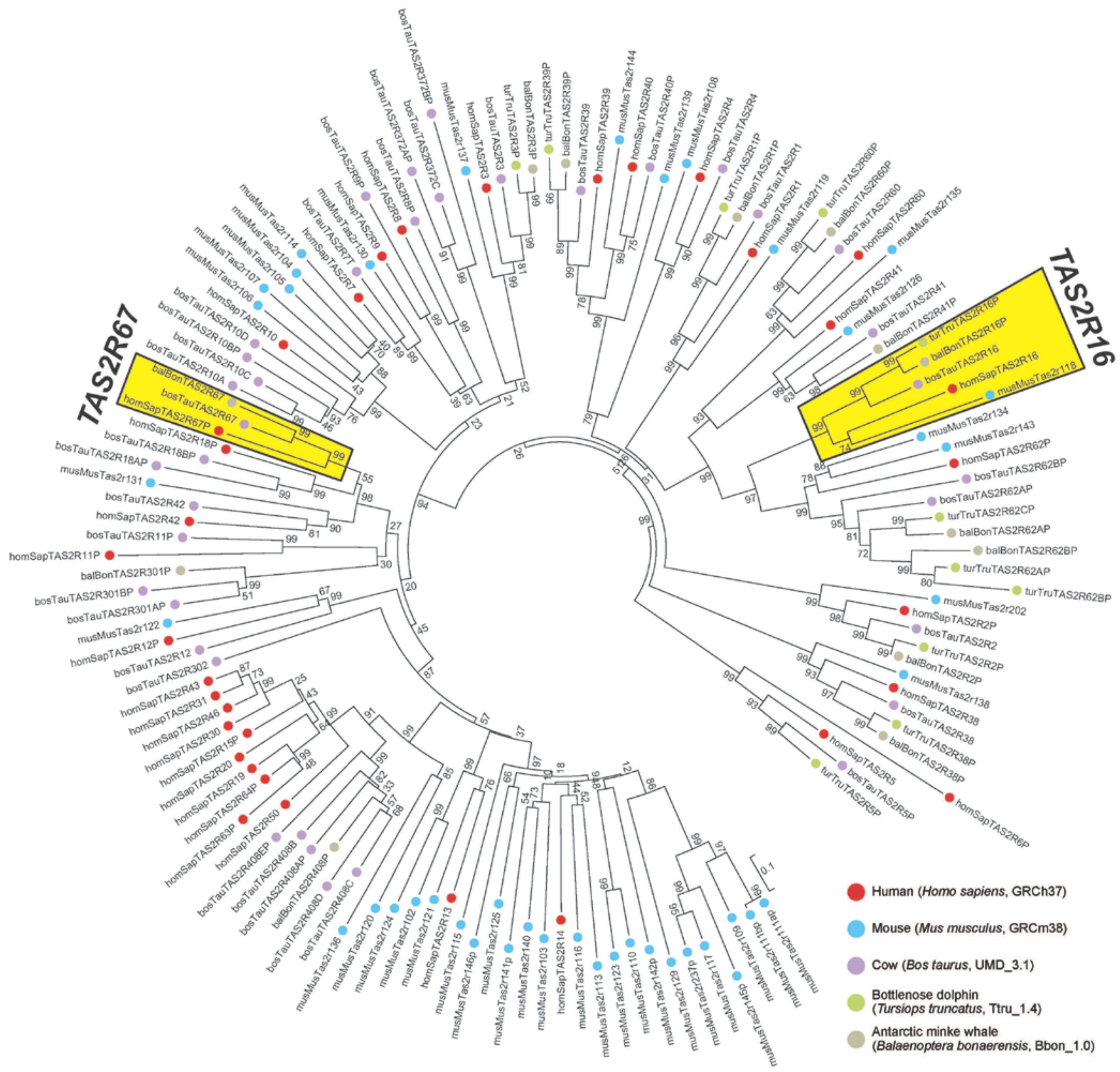

Figure S8. A NJ tree of *TAS2R* genes in five mammals. This tree was based on a multiple alignment of deduced amino acid sequences of all *TAS2R*s annotated in this study, including truncated genes and pseudogenes. Human and mouse TAS2R sequences annotated by Hayakawa et al.[20] were added. *TAS2R16* and *TAS2R67* subtrees are highlighted. The evolutionary distance was calculated for each sequence pair (the pairwise-deletion option) in amino acid sequences based on a Poisson correction distance matrix. Bootstrap values were obtained by 1000 resamplings.

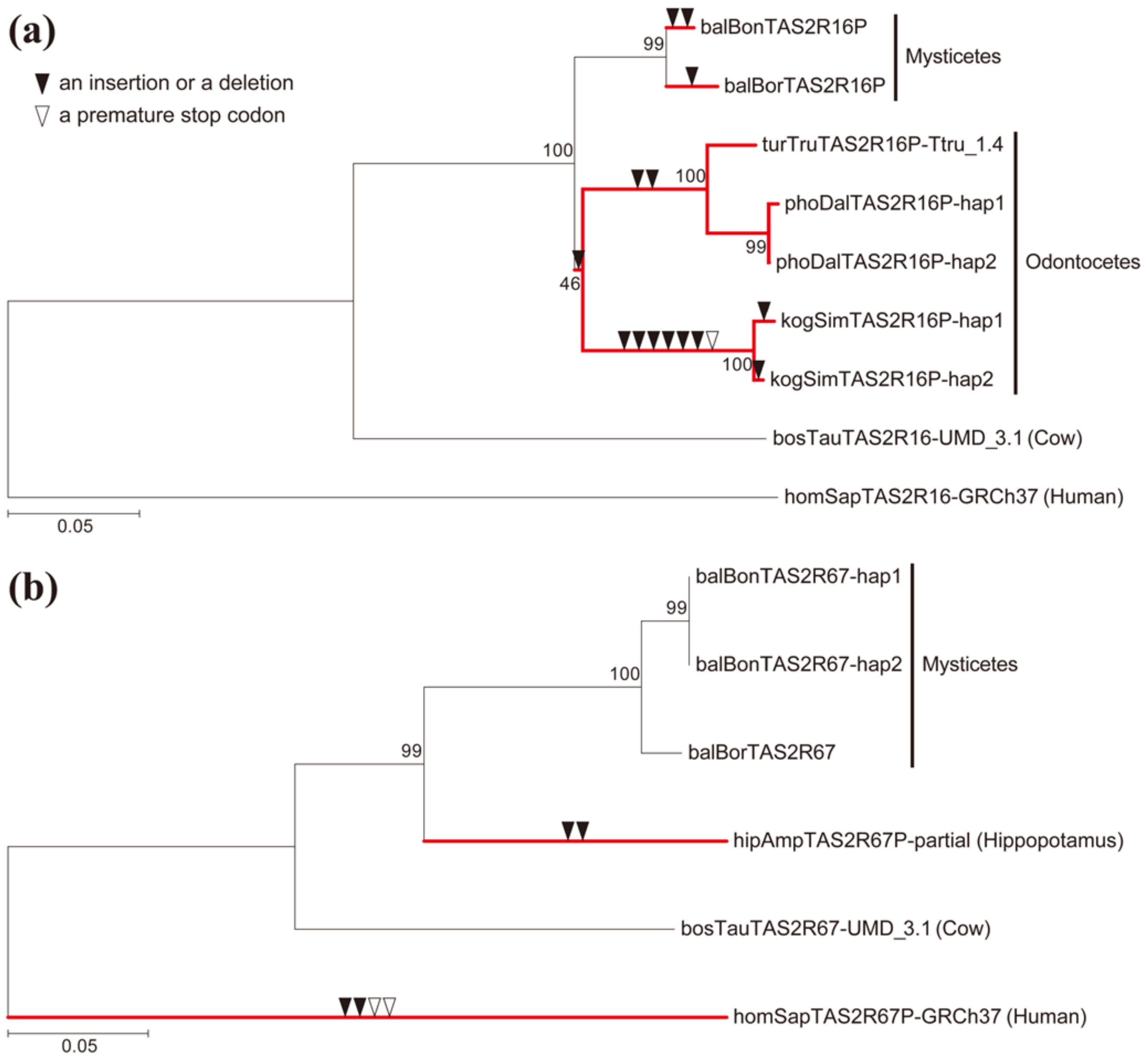

Figure S9. Pseudogenization events of *TAS2R16* (a)/ *TAS2R67* (b) gene in cetartiodactyls. Gene trees are inferred by NJ method based on a Poisson correction distance matrix (Gap sites were excluded from the calculation). Bootstrap values were obtained by 1000 resamplings. Pseudogenization events are indicated by black (frame shift mutations) or white (premature stop codons) triangles. Species name and accession no. of each gene were shown in Table S7. Red branches indicate possession of pseudogenes.

SI References

1. Lohse M, Bolger AM, Nagel A, Fernie AR, Lunn JE, Stitt M, Usadel B: **RobiNA: a user-friendly, integrated software solution for RNA-Seq-based transcriptomics.** *Nucleic Acids Res* 2012, **40:**W622-W627.
2. Luo R, Liu B, Xie Y, Li Z, Huang W, Yuan J, He G, Chen Y, Pan Q, Liu Y: **SOAPdenovo2: an empirically improved memory-efficient short-read *de novo* assembler.** *GigaScience* 2012, **1:**1-6.
3. Kajitani R, Toshimoto K, Noguchi H, Toyoda A, Ogura Y, Okuno M, Yabana M, Harada M, Nagayasu E, Maruyama H, et al: **Efficient de novo assembly of highly heterozygous genomes from whole-genome shotgun short reads.** *Genome Res* 2014, **24:**1384-1395.
4. Yim H-S, Cho YS, Guang X, Kang SG, Jeong J-Y, Cha S-S, Oh H-M, Lee J-H, Yang EC, Kwon KK, et al: **Minke whale genome and aquatic adaptation in cetaceans.** *Nat Genet* 2014, **46:**88-92.
5. Katoh K, Misawa K, Kuma K, Miyata T: **MAFFT: a novel method for rapid multiple sequence alignment based on fast Fourier transform.** *Nucleic Acids Res* 2002, **30:**3059-3066.
6. Katoh K, Kuma K, Toh H, Miyata T: **MAFFT version 5: improvement in accuracy of multiple sequence alignment.** *Nucleic Acids Res* 2005, **33:**511-518.
7. Yang Z: **PAML 4: phylogenetic analysis by maximum likelihood.** *Mol Biol Evol* 2007, **24:**1586-1591.
8. Zhang J, Kumar S, Nei M: **Small-sample tests of episodic adaptive evolution: a case study of primate lysozymes.** *Mol Biol Evol* 1997, **14:**1335-1338.
9. Nei M, Gojobori T: **Simple methods for estimating the numbers of synonymous and nonsynonymous nucleotide substitutions.** *Mol Biol Evol* 1986, **3:**418-426.
10. Yang Z, Kumar S, Nei M: **A new method of inference of ancestral nucleotide and amino acid sequences.** *Genetics* 1995, **141:**1641-1650.
11. Saitou N, Nei M: **The neighbor-joining method: a new method for reconstructing phylogenetic trees.** *Mol Biol Evol* 1987, **4:**406-425.
12. Tamura K, Peterson D, Peterson N, Stecher G, Nei M, Kumar S: **MEGA5: molecular evolutionary genetics analysis using maximum likelihood, evolutionary distance, and maximum parsimony methods.** *Mol Biol Evol* 2011, **28:**2731-2739.
13. Hashiguchi Y, Nishida M: **Evolution of trace amine associated receptor (TAAR) gene family in vertebrates: lineage-specific expansions and degradations of a second class of vertebrate chemosensory receptors expressed in the olfactory epithelium.** *Mol Biol Evol* 2007, **24:**2099-2107.
14. Niimura Y, Nei M: **Evolutionary dynamics of olfactory receptor genes in fishes and tetrapods.** *Proc Natl Acad Sci U S A* 2005, **102:**6039-6044.
15. Xu LL, Stackhouse BG, Florence K, Zhang W, Shanmugam N, Sesterhenn IA, Zou Z, Srikantan V, Augustus M, Roschke V: **PSGR, a novel prostate-specific gene with homology to a G protein-coupled receptor, is overexpressed in prostate cancer.** *Cancer Res* 2000, **60:**6568-6572.
16. Weng J, Wang J, Hu X, Wang F, Ittmann M, Liu M: **PSGR2, a novel G-protein coupled receptor, is overexpressed in human prostate cancer.** *Int J Cancer* 2006, **118:**1471-1480.
17. Durzyński Ł, Gaudin J-C, Myga M, Szydłowski J, Goździcka-Józefiak A, Haertlé T: **Olfactory-like receptor cDNAs are present in human lingual cDNA libraries.** *Biochem Biophys Res Commun* 2005, **333:**264-272.
18. Grus WE, Shi P, Zhang YP, Zhang J: **Dramatic variation of the vomeronasal pheromone receptor gene repertoire among five orders of placental and marsupial mammals.** *Proc Natl Acad Sci U S A* 2005, **102:**5767-5772.
19. Jiang P, Josue J, Li X, Glaser D, Li W, Brand JG, Margolskee RF, Reed DR, Beauchamp GK: **Major taste loss in carnivorous mammals.** *Proc Natl Acad Sci U S A* 2012, **109:**4956-4961.
20. Hayakawa T, Suzuki-Hashido N, Matsui A, Go Y: **Frequent Expansions of the Bitter Taste Receptor Gene Repertoire during Evolution of Mammals in the Euarchontoglires Clade.** *Mol Biol Evol* 2014**:**In press.

21. Kishida T, Kubota S, Shirayama Y, Fukami H: **The olfactory receptor gene repertoires in secondary-adapted marine vertebrates: evidence for reduction of the functional proportions in cetaceans.** *Biol Lett* 2007, **3:**428-430.

Accession numbers

Accession number of each gene sequenced on an automated sequencer (ABI3130, Applied Biosystems) was shown in Table S7.

Table S7. Sequencing results, gene names and accession numbers of *GNAT3* and taste receptor genes. Pseudogenes are indicated with underlines.

	Species	*GNAT3*	*TAS1R2*	*TAS2R16*	*TAS2R67*
Mysticeti	Antarctic minke whale *Balaenoptera bonaerensis*[a]	*balBonGNAT3-hap1* (AB897678) *balBonGNAT3-hap2* (AB897679)	*balBonTAS1R2* (AB897658)	*balBonTAS2R16P* (AB897664)	*balBonTAS2R67-hap1* (AB897672) *balBonTAS2R67-hap2* (AB897673)
Mysticeti	sei whale *Balaenoptera borealis*[b]	*balBorGNAT3* (AB897680)	*balBorTAS1R2* (AB897659)	*balBorTAS2R16P* (AB897665)	*balBorTAS2R67* (AB897674)
Odontoceti	Dall's porpoise *Phocoenoides dalli*[c]	*phoDalGNAT3* (AB897682)	not amplified	*phoDalTAS2R16P-hap1* (AB897668) *phoDalTAS2R16P-hap2* (AB897669)	not amplified
Odontoceti	dwarf sperm whale *Kogia sima*[c]	not amplified	*kogSimTAS1R2-hap1* (AB897662) *kogSimTAS1R2-hap2* (AB897663)	*kogSimTAS2R16P-hap1* (AB897670) *kogSimTAS2R16P-hap2* (AB897671)	not amplified
Artyodactyla	hippopotamus *Hippopotamus amphibius*[d]	*hipAmpGNAT3* (AB897683)	not amplified	not amplified	*hipAmpTAS2R67P* (AB897677)

notes: [a]The genomic DNA prepared for whole-genome shotgun sequencing (see §1) was used

[b]Muscle tissue of this whale was purchased from a fish market in Japan

[c]Genomic DNA was prepared in our previous study[21]

[d]Muscle tissue of a hippopotamus was kindly provided by Dr. Takeshi Wada, Osaka Museum of Natural History
